# The use of isCGM leads to marked reduction in severe hypoglycemia requiring emergency medical service or hospital admission and diabetic ketoacidosis in adult type 1 diabetes patients

**DOI:** 10.1007/s00592-023-02079-y

**Published:** 2023-03-28

**Authors:** Jyrki Mustonen, Päivi Rautiainen, Marja-Leena Lamidi, Piia Lavikainen, Janne Martikainen, Tiina Laatikainen

**Affiliations:** 1 Department of Internal Medicine, Joint Municipal Authority for North Karelia Social and Health Services (Siun Sote), Tikkamäentie 16, 80210 Joensuu, Finland; 2 Institute of Public Health and Clinical Nutrition, University of Eastern Finland, Yliopistonranta 1, 70210 Kuopio, Finland; 3 School of Pharmacy, University of Eastern Finland, Yliopistonranta 1, 70210 Kuopio, Finland; 4 Department of Public Health and Welfare, National Institute for Health and Welfare (THL), Helsinki, Finland

**Keywords:** isCGM, Glucose monitor, Type 1 diabetes, Hypoglycemia, Diabetic ketoacidosis

## Abstract

**Aims:**

To determine the effect of the use of intermittently scanned continuous glucose monitoring (isCGM) on acute diabetes-related complications in adult type 1 diabetes patients.

**Methods:**

Six hundred and forty-two adult type 1 diabetes patients with isCGM were identified from electronic health records in Siun sote region in Eastern Finland. A retrospective real-world analysis was conducted combining hospital admission and prehospital emergency service data to compare incidences of hypoglycemia requiring emergency medical support (EMS) involvement or hospital admission and diabetic ketoacidosis (DKA) before and after the start of isCGM. Data were collected from January 2015 to April 2020. Primary outcome was the rate of hypoglycemia requiring EMS involvement or hospital admission and DKA events. HbA1c was recorded at the start of isCGM and was compared with the last known HbA1c during the use of isCGM. The isCGM used in the study did not contain alarm functions.

**Results:**

Altogether 220 hypoglycemic events were identified during the study period. Incidence rate of hypoglycemic events decreased after the start of isCGM (72 events, incidence rate 50 events/1000 person-years) compared with the time before the start (148 events, incidence rate 76 events/1000 person-years) (*p* = 0.043). The incidence rate of DKA decreased after the start of isCGM compared with time before isCGM use (4 and 15 events/1000 person-years, respectively; *p* = 0.002). The change in mean HbA1c was − 0.28% (− 3.1 mmol/mol) between baseline and the last HbA1c measurement (*p* < 0.001).

**Conclusions:**

In addition to lowering HbA1c in type 1 diabetes patients, isCGM is also effective in preventing acute diabetes-related complications such as hypoglycemia requiring EMS involvement or hospital admission and DKA.

## Introduction

Hypoglycemia and diabetic ketoacidosis (DKA) are acute and potentially life-threatening complications of diabetes. When aiming to achieve tight glycemic control to avoid chronic complications, the rate of hypoglycemia is often elevated [1]. ADA workgroup on hypoglycemia defined hypoglycemia to cover all episodes of an abnormally low plasma glucose concentration that expose the individual to potential harm [2]. Hypoglycemia can be divided as mild (self-treated) or severe (requiring external help). In the Danish–British multicenter study, type 1 diabetes patients experienced on average two mild hypoglycemic events weekly and 37% reported severe hypoglycemia in a year [3].

DKA is characterized by lack of insulin action and hyperglycemia. DKA most often affects patients with type 1 diabetes. According to a systematic literature review, incidence of DKA varies from 0 to 263 per 1000 patient-years [4]. Being a severe condition, DKA is usually treated in hospital in contrast to hypoglycemia. An observational study showed that hospital admissions represent the minority of severe hypoglycemic events (37.9%), and many of the hypoglycemia episodes are treated at home by emergency medical assistance of ambulance services [5].

Achieving good glycemic control has been traditionally done by self-monitoring blood glucose (SMBG). In the past few years, new technology is introduced to replace SMBG since there is growing evidence that both real-time continuous glucose monitoring (rtCGM) [6] and intermittently scanned continuous glucose monitoring (isCGM) [7] improve treatment outcomes (in terms of lower HbA1c levels) in type 1 diabetes patients.

Real-world studies also suggest lower rates of DKA after initiation of rtCGM [8] or isCGM [9, 10]. Use of rtCGM reduced severe hypoglycemic events in RCT settings compared with self-monitoring blood glucose (SMBG) [11, 12]. However, results are contradictory whether isCGM reduces severe hypoglycemic events [9, 13] which can be due to the nature of the studies and classification of severe hypoglycemia.

Commonly, real-world studies rely on hospital admission [9] or questionnaire data [13] which can lead to possible underestimation of severe hypoglycemia and may dilute potential effect of isCGM if patients are not transported to hospital or do not recall all hypoglycemic events. To be more objective, in the present study, we combined electronic health record-based hospital admission and the use of emergency medical service data in isCGM users, aiming to investigate potential benefit of isCGM to reduce acute complications in type 1 diabetes patients.

## Methods

### Design and participants

The present retrospective study was carried out in the area of the Joint Municipal Authority for North Karelia Social and Health Services (Siun sote), which provides social service, healthcare services (both primary and specialized health care), and emergency service for the inhabitants (*n* = 166,400) living in its catchment area. FreeStyle Libre isCGM (later isCGM) was introduced to patients in 2016. From 2016 to 2020, Siun sote-funded isCGM was limited to type 1 diabetes patients fulfilling special criteria as described in our previous article [14]. These criteria included (1) suboptimal HbA1c despite optimized treatment with repeated hypoglycemic events, (2) planning to become or being pregnant, (3) fear of hypoglycemia, (4) fear of needles, leading to inability to perform SMBG, (5) end-stage renal disease and dialysis treatment, (6) inability to execute SMBG because of amputation or disability, and (7) difficulties in carrying out SMBG because of occupation. If at least one of these criteria was fulfilled, isCGM could be initiated.

**Fig. 1:**
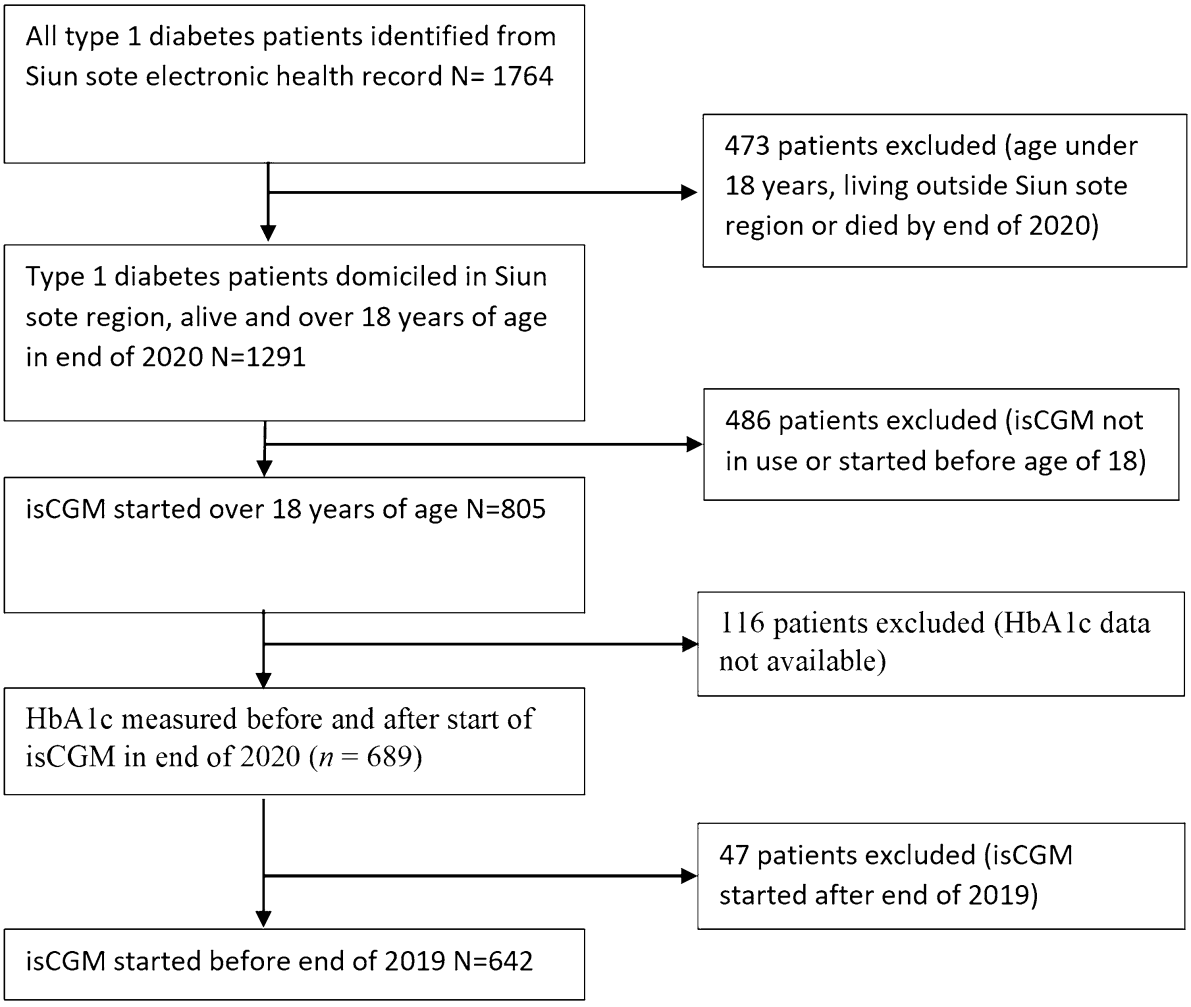
Patient selection

Identification of patients with type 1 diabetes in the Siun sote region was done using ICD-10 codes (E10.1–E10.9), and the data were collected from the electronic health records as described in our previous article [15]. A total of 1764 patients with type 1 diabetes were identified, of whom 805 patients were domiciled in the Siun sote region, were alive at the end of 2020, and were 18 years or more at the time of the start of isCGM. Patients who were continuously using isCGM were identified from the electronic health records. The starting date of continuous use of isCGM was identified from the electronic health records, and if the use of isCGM had ended, the registered end date was also collected. Patients who started isCGM prior to the end of 2019 were included in the analysis (Fig. 1).

Information on HbA1c levels and admissions to hospital for severe hypoglycemia (ICD code E10.00) and DKA (ICD code E10.1) was collected from the electronic health records. In addition to acute complications that were retrieved from the EHR, severe hypoglycemic events requiring emergency medical services (EMS) were retrieved from the records of the ambulance service. All visits of ambulance service to the patients in the study population were manually validated by an experienced diabetologist (JMu) to identify cases of severe hypoglycemia requiring EMS service (later hypoglycemic event) and were included in the analysis if one or more following conditions were satisfied: (1) First measured plasma glucose by paramedics was 70 mg/dL (3.9 mmol/l) or less, (2) primary unconsciousness withdrawn by glucose administration, (3) glucose administration by paramedics, or (4) glucose administration by another person. If the patient was transported to hospital by emergency medical service, only one event was reported from either EHR or records of the ambulance service. Data were collected from the beginning of 2015 to the end of April 2020. EMS electronic database was altered in the end of April 2020, so it was chosen as an endpoint of the study. Self-reported hypoglycemia was not registered and thus was not included in the analysis. DKA event at the point of diabetes diagnosis was excluded from the analysis. The type of isCGM in this study was FreeStyle Libre 1 which does not have alarm functions, and it requires user action to get the real-time glucose information. FreeStyle Libre 2 system which has optional alarms was not used in this study because it was not available in Siun sote region during the study period.

Before initiation of isCGM, patients received 1‒2-hour introduction from a nurse specialized in diabetes care. No special interventions were introduced to the patients after starting isCGM, and they received standard clinical care during the time using isCGM usually consisting of 1–2 visits to a nurse or a diabetologist annually. The data from isCGM were used to optimize treatment during the visits. For this study, sensor metrics were not available and were not used in the analysis.

Due to the retrospective register-based nature of the study, the approval to conduct the study was acquired from the Institutional Review Board of Siun sote.

### Statistical analyses

Descriptive statistics, such as means, medians, and standard deviations, were used to describe the data. Incidence rate, i.e., number of events divided by 1000 person-years, was used for the occurrence of hypoglycemic events and DKA before and after the start of isCGM. Baseline of the analysis was the time of isCGM initiation. Dependent samples t test was used to test difference in HbA1c values between baseline and the last measurement during the follow-up. Poisson’s regression with generalized estimating equation (GEE) was used to examine differences in incidence rates before and after initiation of isCGM. Number of events was used as an independent variable with natural logarithm of person-years as an offset variable, and subject id was used as a subject effect variable as each individual served as their own control. Also, gender, classified HbA1c, and classified age were added to the model separately with time and their interaction to see whether the time effect was different in separate classes. Subgroup analyses were done as post hoc analyses from these models when there was interaction between time and other explanatory factors. HbA1c classes < 7%, 7–9%, and > 9% (< 53, 53–75, and > 75 mmol/mol) were used in the subgroup analyses. The R language and environment for statistical computing (version 4.0.3) and IBM SPSS Statistics for Widows (version 27.0) were used in statistical analyses. *P* values less than 0.05 were regarded as statistically significant, and 95% confidence intervals (Cis) were used for statistics.

## Results

A total of 642 patients met the inclusion criteria. Baseline characteristics are displayed in Table 1. Mean age was 47 years. HbA1c level at the start of isCGM was 8.7% (71.6 mmol/mol). Median follow-up before and after the initiation of isCGM was 2.87 and 2.45 years, respectively. Six hundred and thirty-one patients had at least one HbA1c measurement done after the start of isCGM. Median time from the start of isCGM to the last HbA1c measurement was 2.05 years. The change in mean HbA1c levels was − 0.28% (− 3.1 mmol/mol) between baseline and the last HbA1c measurement during the follow-up (*p* < 0.001). 8.7% of the patients had continuous subcutaneous insulin infusion (CSII) at the start of isCGM.

**Table 1:** Characteristics of patients at the time of isCGM initiation (*n* = 642)

Age (years ± SD)	47.0 ± 16.2
Age min–max Duration of diabetes in years*, median (min–max)	18.1–83.4 23.7 (0.1–69.0)
HbA1c % ± SD (mmol/mol ± SD)	8.7 ± 1.5 (71.6 ± 15.9)
HbA1c % (mmol/mol) min–max	5.2–15.2 (33.0–143.0)
HbA1c < 7% (< 53 mmol/mol), *n* (%)	57 (8.9%)
*Gender, n (%)*	
Men	349 (54.4%)
Women	293 (45.6%)
Insulin therapy**, n (%)	
CSII	56 (8.7%)
MDI	586 (90.3%)
*isCGM use, years median (min*–*max)*	
Before first event ***	2.87 (1.4–5.0)
After first event ***	2.45 (0.3–3.9)

*isCGM* intermittently scanned continuous glucose monitor; *CSII* continuous subcutaneous insulin infusion; *MDI* multiple daily injections

^*^From diagnosis to start of isCGM

^**^Prior to start of isCGM

^***^Hypoglycemic event

**Fig. 2:**
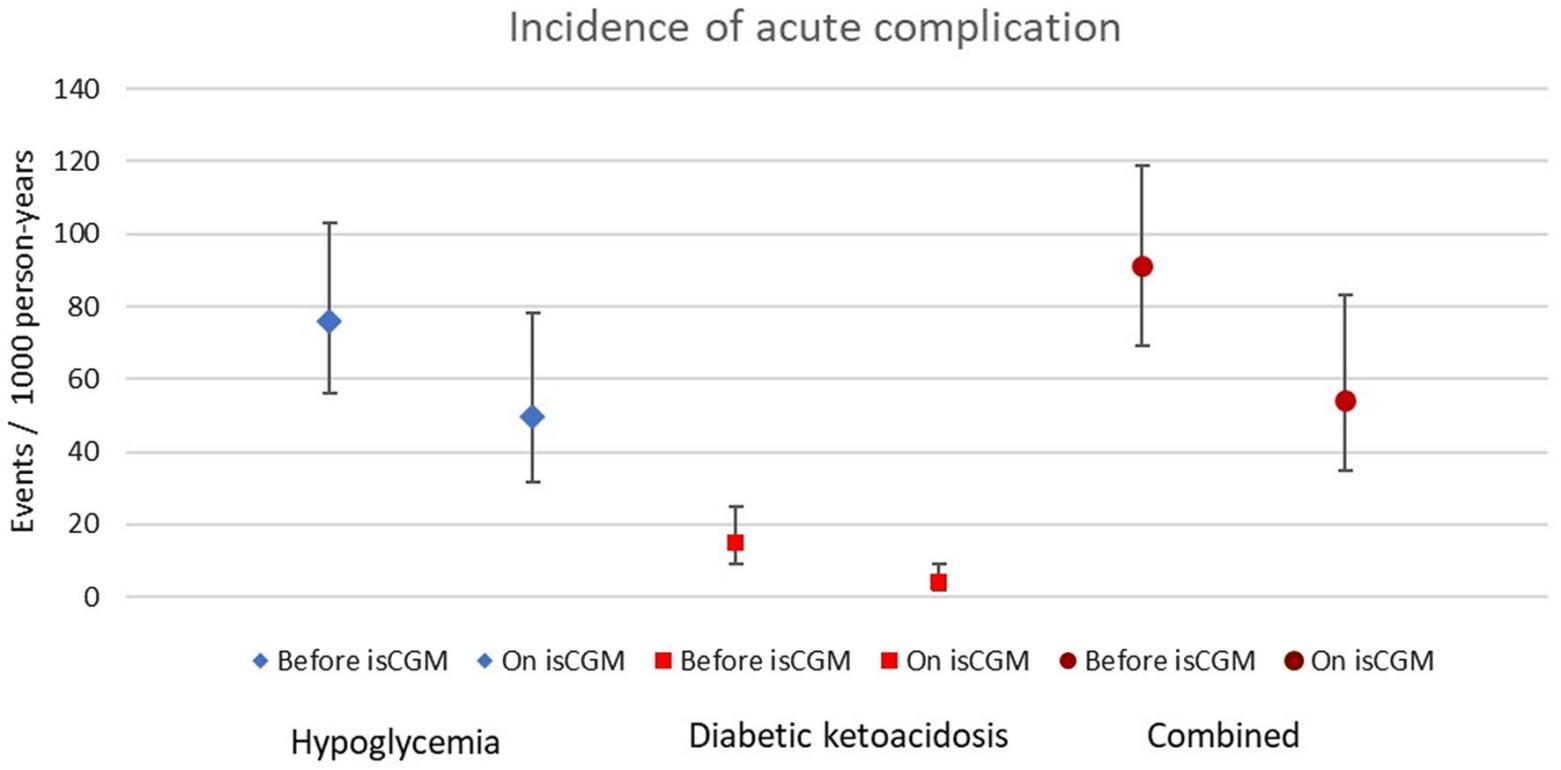
Incidence with 95% CI of acute complication

Altogether 213 hypoglycemic events requiring EMS were identified, and in 51 events (23.8%), patient was transported to the emergency department. In addition, 7 patients were admitted to hospital due to hypoglycemia without EMS involvement. Eighty-three patients encountered hypoglycemic event before isCGM initiation and 43 patients after the start of isCGM, and the frequencies of hypoglycemic events per patient varied from 0 to 11 and from 0 to 12, respectively. Of these 220 hypoglycemic events, 148 occurred prior to isCGM start and 72 after the start of isCGM, incidence rate being significantly lower after the start: 76 and 50 events/1000 person-years, respectively (*p* = 0.043) (Fig. 2).

In the study cohort, a total of 35 ketoacidosis events were identified. From these, 29 occurred prior to the start of isCGM and 6 after start of isCGM representing significant reduction in incidence from 15 to 4 events/1000 person-years, respectively (*p* = 0.002) (Fig. 1).

When hypoglycemic and DKA events were combined as an acute complication, the reduction in these events was significant (177 vs 78 events; 90 and 54 events/1000 person-years when comparing events before and after isCGM start) (*p* = 0.008) (Fig. 1).

In subgroup analysis stratified by baseline HbA1c levels (HbA1c < 7%, 7–9%, and > 9% (< 53, 53–75, and > 75 mmol/mol)), the reduction in hypoglycemic events after the start of isCGM did not reach statistical significance. Patients with highest HbA1c (> 9% (> 75 mmol/mol)) showed a trend in lower rate of hypoglycemic events although it did not reach statistical significance (*p* = 0.064). Patients whose HbA1c level was > 9% (> 75 mmol/mol) showed significant reduction in DKA events after the start of isCGM (31 vs 5 events/1000 person-years before and after the start of isCGM, respectively) (*p* = 0.002) (Table 2).

**Table 2:** Incidence of acute complications

Acute complication	Events/1000 PY (number of events)		
	Prior to isCGM	After start of isCGM	*P* value
*Severe hypoglycemia*			
All patients (*n* = 642)	76(148)	50(72)	**0.043**
HbA1c subgroups			
HbA1c < 7% (*n* = 57)	111(20)	57(7)	0.167
HbA1c (7%, 9%) (*n* = 351)	73(80)	59(45)	0.419
HbA1c > 9% (*n* = 234)	71(48)	35(20)	0.064
*Diabetic ketoacidosis (DKA)*			
All patients *n* = 642	15(29)	4(6)	**0.002**
HbA1c subgroups			
HbA1c < 7% (*n* = 57)	0	0	NA
HbA1c (7%, 9%) (*n* = 351)	7(8)	4(3)	0.289
HbA1c > 9% (*n* = 234)	31(21)	5(3)	**0.002**
*Severe hypoglycemia or DKA*			
All patients (*n* = 642)	90(177)	54(78)	**0.009**
HbA1c subgroups			
HbA1c <7% (*n* = 57)	111(20)	57(7)	0.167
HbA1c (7%, 9%) (*n* = 351)	80(88)	63(48)	0.329
HbA1c > 9% (*n* = 234)	102(69)	41(23)	**0.005**

*isCGM* intermittently scanned continuous glucose monitor**;** *DKA* diabetic ketoacidosis; *PY* person-years

Bold values indicates the statistically significant

Age at the start of isCGM was not correlated with the change in hypoglycemic or DKA events. Furthermore, there was no difference between genders in acute complications. If the patient had CSII at the onset of isCGM, there was not a significant change in hypoglycemic (*p* = 0.330) or ketoacidosis events (*p* = 0.670).

## Discussion

In the present study, we observed a significant reduction in severe hypoglycemia requiring EMS involvement or hospital admission and DKA after the initiation of isCGM. Based on our knowledge, this is the first study combining prehospital emergency medical support and hospital admission data directly from electronic health records (instead of questionnaires) to identify severe hypoglycemic and DKA events in type 1 diabetes patients using isCGM. In addition, our study demonstrated significant reductions in both severe hypoglycemia requiring EMS or hospital admission and hospitalizations for DKA after start of isCGM in a real-world setting. Also, the significant reduction of HbA1c is in line with previous RCTs [16] and real-world studies [7, 14, 17], as well as a recent meta-analysis [18], showing that isCGM is efficient in lowering HbA1c in patients with type 1 diabetes although our findings were modest compared to the findings from Evans et al. (− 0.28% (− 3.1 mmol/mol) vs − 0.56% (− 6.1 mmol/mol)) [18] which may be due to the longer follow-up in our study.

According to our findings, the use of isCGM significantly lowers also the rate of severe hypoglycemic events requiring EMS involvement or hospital admission. In a RCT setting [16], isCGM users spend less time in hypoglycemic range, and in real-world setting, Nathanson et al. [13] found that there were less self-reported hypoglycemic events and marginally but significantly lower HbA1c when comparing isCGM users to control using SMBG in a 2-year follow-up. In a nationwide audit to clinicians in the UK [19], the use of isCGM significantly reduced absolute number of paramedic callouts and hospital admissions due to hypoglycemia when comparing 12 months before starting isCGM and seven-and-a-half-month follow-up. In addition, a real-world study showed that the number of hospital admissions due to severe hypoglycemia reduced significantly after start of isCGM without deterioration of HbA1c levels [20]. These findings are in line with our study. However, there are also studies that failed to demonstrate a positive effect of isCGM in preventing patients from severe hypoglycemia. For example, a previous prospective, open-label RCT did not found a significant difference in severe hypoglycemia between isCGM and SMBG in a 6-month follow-up [21]. A prospective observational study by Stimson et al. showed an increase in hypoglycemia admissions after initiation of isCGM during 12-month follow-up [22]. In a RCT comparing rtCGM and isCGM, the rate of self-reported severe hypoglycemic events was lower in the rtCGM group but not in the isCGM group in 6-month follow-up [23].

One possible reason for conflicting findings concerning severe hypoglycemia in isCGM users could be that patients do not recall or do not want to report severe hypoglycemic events. Pedersen-Bjergaard et al. showed that changes in EU driver’s licence legislation led to significant reduction in self-reporting severe hypoglycemic events in type 1 diabetes patients [24]. Another possible explanation is that hospital admissions represent only a part of severe hypoglycemia. A study by Villani et al. [5] found that 38% of patients requiring emergency medical service were transported to hospital which was more frequent than in our study. In our study, only 23.8% of patients requiring EMS for hypoglycemia were transported to hospital, which is similar to the proportions observed in previous studies [25, 26]. When patients are not admitted to a hospital, the possible beneficial effect of isCGM can be underestimated in studies relying on hospital admission data only.

The role of sensor alarm function has been discussed when comparing rtCGM and isCGM [23]. RtCGM devices have had alarm functions for a while, but first generation of isCGM does not provide alarms. Our study does not support an assumption that the alarm function is the key factor in reducing severe hypoglycemia because isCGM in our study was the FreeStyle Libre 1 without alarms. This might be because some patients suffer from alarm fatigue as described by Shivers et al. [27] and turn off other than mandatory hypoglycemic alarms of rtCGM.

Our study showed a significant reduction in the rate of DKA. Real-world studies have also shown that the use of both rtCGM^8^ and isCGM [7, 9, 28] reduces admission due to DKA. Tyndall et al. showed that the start of isCGM reduces significantly hospital admissions caused by DKA in 900 type 1 diabetes patients in a median follow-up of 245 days although the rate of DKA was small (10 events before and 2 after start of isCGM) [7]. In a nationwide cohort, France Roussel et al. found that the use of isCGM led to significantly lower incidence of DKA [9]. They identified a total of 33,165 patients with type 1 diabetes and hospitalizations because DKA fell 56.2% in follow-up of 12 months in comparison with 12 months before the start of isCGM. This effect was maintained over two-year period in a follow-up study of the original analysis [29]. In a study of 46 type 1 diabetes patients, Hayek et al. demonstrated that the use of isCGM was associated with a significant reduction in DKA [28]. These findings are in line with our data. Although the number of DKA events was small in our study, the reduction is significant. In FUTURE study by Charleer et al., the use of isCGM did not lead to significant reduction in hospital admissions due to DKA [20]. These findings conflict with our present findings. Weinstock et al. found that the frequency of DKA is increased with higher HbA1c levels [30]. In our study, HbA1c level at the start of isCGM (8.7% or 71.6 mmol/mol) was higher than in the FUTURE study where the HbA1c at the baseline was 7.8% (62 mmol/mol) which can affect the results. This effect might be due to the intensification of insulin therapy after the start of isCGM as the group with highest HbA1c levels showed the greatest drop in ketoacidosis admissions. The reduction of DKA is important because it has been shown that recurrent DKA is associated with increased mortality [31]. The dispersion of results concerning isCGM and acute diabetes-related complications can be because starting criteria for isCGM differ between different study populations, and selection bias can affect the results also in our study.

The strength of the present study is that it combines EMS and hospital electronic health records to cover both prehospital and hospital care of acute diabetes-related complications. As multiple reasons can affect studies relying only on hospital admission and/or questionnaire data concerning severe hypoglycemia, some events could be therefore missed from the analysis and the potential beneficial effect can be diluted. It is also important to point out that we were able to exclude ketoacidosis events at the point of diabetes diagnosis.

Our study has also several limitations. As being entirely register-based study, it lacks control population and the confounding effect of other procedures to reduce acute events cannot be ruled out. Due to the retrospective real-world setting, the EMS and hospital admissions data collection time is not identical before and after starting isCGM. Our study population is also relatively small compared with large register studies and due to this subgroup analysis may lack power to show beneficial effect of isCGM. As the present study was entirely EHR based, we do not have information on the exact indication of use of isCGM in each individual, exact sensor metrics such as active time of sensor use or time in range. The use of only first-generation isCGM can also be seen as a limitation as technology is moving toward rtCGM with alarm functions such as FreeStyle Libre 3. In further studies, it can be difficult to show significant drop in acute complications as more and more type 1 diabetes patients are on rtCGM or isCGM instead of SMBG already.

## Conclusions

In summary, this study adds important information of isCGM use not only to lower HbA1c but also to reduce significantly severe hypoglycemia requiring EMS or hospital admission and hospitalizations of DKA. It is important to notice that reduction of these acute complications does not require alarm functions of isCGM. Further studies are needed to show the overall benefit of isCGM use in long term.

## Data Availability

M.D. Jyrki Mustonen is the guarantor of this work and, as such, had full access to all the data in the study and takes responsibility for the integrity of the data and the accuracy of the data analysis.

## Abbreviations

ADA: American Diabetes Association
CSII: Continuous subcutaneous insulin infusion
DKA: Diabetic ketoacidosis
EMS: Emergency medical support
isCGM: Intermittently scanned continuous glucose monitoring
rtCGM: Real-time continuous glucose monitoring
SMBG: Self-monitoring blood glucose
